# Mental health conditions in people searching for aesthetic treatments: The importance of early detection

**DOI:** 10.1192/j.eurpsy.2021.1224

**Published:** 2021-08-13

**Authors:** F. Gonçalves Viegas, I. Figueiredo, F. Ferreira, A. Lourenço

**Affiliations:** Departamento De Saúde Mental, Hospital Prof Dr Fernando Fonseca, Amadora, Portugal

**Keywords:** psychiatric conditions, aesthetic, early detection, prevention

## Abstract

**Introduction:**

It’s established that aesthetic dysfunctions can be associated with psychiatric conditions. In present times, considering the exponential growth of minimally invasive and accessible techniques, alongside with ideals of beauty present in everyday life through exposure in social media, the importance of early detection of mental illness and its impact on the respective outcome should be emphasized.

**Objectives:**

To review evidence regarding psychiatric disorders in people searching for aesthetic treatments and their impact on the outcome.

**Methods:**

Literature review using Medline database.

**Results:**

Around 50% of individuals seeking aesthetic procedures fulfill the diagnostic criteria for psychiatric disorders. The prevalence of Body Dysmorphic Disorder (BDD) can vary from 5-15%, with some studies showing a prevalence of more than 50%. Patients with heightened BDD symptoms are less satisfied with the outcomes of aesthetic procedures which could result in exacerbation of said symptoms. With regards to eating disorders, evidence suggests the initial satisfaction following aesthetic procedures, when observed, is usually transitory, not leading to long-term changes in self-perception relating to body image, nor improving prognosis or quality of life. There’s also some evidence suggesting that personality disorders may be a predictor of poor satisfaction with the results of aesthetic treatments.

**Conclusions:**

Awareness should be raised in this matter, since psychiatric conditions are more common in patients seeking aesthetic treatments and early identification can lead to a better prognosis by providing patients with the mental health treatment they need; this could also reduce the probability of dissatisfaction and subsequent aggravation of psychiatric symptoms following aesthetic interventions.

